# Hahnemann’s Therapeutic Hints

**Published:** 1895-11

**Authors:** 


HAHNEMANN'S THERAPEUTIC HINTS. Collected and arranged by R. E. Dudgeon, M. D. London: E. Gould & Son, 59 Moorgate Street, E. C. 1894.

This little book of sixty pages is a repertory of the clinical experience scattered through the works of Hahnemann.

In his introduction to the book the author says: "It has always been a matter of regret to his disciples that Hahnemann did not publish any systematic work on his therapeutics. His unrivaled powers of observation, his intimate acquaintance with the actions of medicines, and his vast experience would have enabled him to produce a manual of inestimable value to the practitioner. But though Hahnemann has not written any complete work on his clinical experiences, his writings are by no means destitute of valuable indications for the remedial employment of many of the medicines whose pathogenetic properties he had investigated. The great number of these indications is to be found in the introductory observations prefixed to most of the pathogeneses of the medicines contained in the *Chronic Diseases*."

In accordance with the foregoing statement the book before us contains these indications in the form of a repertory. The arrangement is by regional chapters, as in most other repertories, and these again subdivided into the various headings that are needful to locate a symptom. The book is undoubtedly a help in the selection of the remedy, and it is at the same time a ready means of arriving at Hahnemann's own experience in regard to any sick condition. This feature of it will be invaluable to any practitioner who vividly realizes the immense import of Hahnemann's innovations into the methods of the old school of medicine, and at the same time is sufficiently free from the usual arrogance of reason to recognize that there are many things not dreamed of in his rational philosophy, and so cordially admits the unquestionable merit and high authority of the great discoverer.

